# Public Employment of Physicians

**Published:** 1915-10

**Authors:** 


### Public Employment of Physicians.

Both sociologically, in regard to the tendency to remove medical practice from the field of competitive industry, and economically as a means of employment for an overcrowded profession, the number of physicians publicly employed, is a matter of great interest. Some months ago, we began an inquiry into this matter, expecting that a few letters to central bureaus would easily secure the desired information for the nation, state, county and city. On the contrary, it has been learned that physicians are employed by a large variety of subdivisions of government, in very different numbers, and that there is no single bureau from which statistics can be obtained for any main division of the government of this country.

It has been assumed that $1200 is a living wage, and, indeed, this amount is probably fully equal to the average net professional income of physicians in private practice. This amount has, therefore been taken as the minimum salary in compiling the following statistics, except that in the case of resident physicians, salaries of $1000 plus board, have been considered equivalent. In addition to the professional employment thus listed, a survey of appointment lists has shown a surprising number of positions requiring special education in chemistry, bacteriology, sociology and natural sciences, for which physicians would be eligible if they found it desirable to obtain employment. There is also an enormous amount of employment of physicians on fees or small salaries for partial services. For example, the Indian Field Service employs 62 physicians who are in private practice, on salaries averaging $577.66 and the Bureau of Pensions has 4,635 medical examiners paid by fees. Members of examining boards, physicians performing special services for counties, cities and villages, and members of special commissions, internes in various institutions, etc., are quite numerous and are paid, generally speaking, at a much higher rate than the minimum salary of $1200, allowing for the amount of time required or for the fact that internes are obviously compensated in experience and that their earnings in private practice would be very small. No attempt has been made to estimate the amount of employment analogous in its economic features, to that by divisions of the government—as by insurance companies, mines and manufacturing industries, private hospitals and endowed philanthropic institutions.

Employment by U. S.

| | | |
|---|---|---|
| Army, active | 424 | |
| " retired | 97 | |
| " reserve and cont. | 13 | Total . . 534 |
| Navy, active | 380 | |
| " retired | 105 | " . . 485 |
| Pub. Health commissioned | 186 | |
| " " contract | 71 | " . . 527 |
| Indian Field Service | | 102 |
| Pensions | | 19 |
| Pánama Canal | | 37 |
| Miscellaneous | | 100 |
| | | 1534 |

(Estimate from announcements of positions open).

Employment by N. Y. State.

| | |
|---|---|
| Dept. of Health | 27 |
| Port of N. Y. | 12 |
| Hospital Commission | 5 |
| State Hospitals, staff | 167 |
| " " internes | 18 |
| Inst. Malig. Dis. | 5 |
| Psychiatric Inst. | 3 |
| " " interne | 1 |
| Saratoga Reservation | 1 |
| Workmen's Compensation | 3 |
| Prisons, Asylums, etc. | 37 |
| | 279 |

Employment by Erie Co. . . . . 4

Employment by Buffalo.

| | |
|---|---|
| Dept. of Health | 22 |
| Police, Fire, etc. | 4 |
| | 26 |

As nearly as can be estimated, these numbers correspond to 1.15% for the country; 2% for the state; 0.5% for the county and 3.7% for the city, a total, not allowing for the successive slight decrease of the basis on which percentages should be computed, of 7.35%. Owing to fluctuations in the number of physicians employed for partial services, and especially when payment is by fees or for temporary contracts, it has not been possible to secure statistics of this nature, except for certain branches of the government. So far as can be estimated from the various statements received, the salaries and fees amounting to less than $1200 per capita per annum correspond to half the minimum salary for at least as many physicians as those employed on salaries of $1200 and upward. Thus, if distributed in larger amounts to fewer men, about 10% of the profession would be supported by the different branches of government, on salaries of $1200 and upward. Of the 7.35% who actually receive such salaries, the average salary is considerably higher, say $1500-$2000. When we consider that the salary is supplemented by office and equipment, clerical help, allowance for transportation and other expenses and that a sufficient force is usually provided for to allow for reasonable vacations, it will readily be appreciated that the salary must be compared with net instead of gross professional earnings in private practice. Thus, to say that 7.35% of the profession are actually supported by the government and that an equivalent of 10% of the profession is thus supported, allowing for partial employment, probably means that considerably more than 10% and possibly as much as 20% of the support of the profession is derived from one branch or another of our government. In other words, we have gone a good deal farther toward the fulfillment of the conception of socialists regarding medical services, than is usually realized .

While considerable time has been spent in the collection of the data presented, they are inevitably far from complete. With the exception of the estimate of 100 physicians employed in various ways by the national government, of whom neither the Civil Service Commission nor any other central bureau has records, errors must be in the direction of omissions while the general tendency is to increase rather than to decrease the positions of public employment. Percentages of physicians employed by the government may, of course, be exaggerated by incompleteness of directory lists. So far as we can judge, however, by checking up such lists for areas with which we are personally familiar, very few omissions of physicians actually in practice occur while, on the other hand, a considerable number of names are included of persons with medical degrees but who are not in practice Thus, we feel confident that our statistics err on the side of conservatism for a double reason.

